# Structures and mechanism of the AUX/LAX transporters involved in auxin import

**DOI:** 10.1038/s41477-025-02056-z

**Published:** 2025-08-04

**Authors:** Kien Lam Ung, Lukas Schulz, Lorena Zuzic, Bjørn Lildal Amsinck, Sarah Koutnik-Abele, Ines Benhammouche, Camilla Gottlieb Andersen, Lynette Nel, Birgit Schiøtt, David L. Stokes, Ulrich Zeno Hammes, Bjørn Panyella Pedersen

**Affiliations:** 1 Department of Molecular Biology and Genetics, Aarhus University, Aarhus, Denmark; 2 Plant Systems Biology, School of Life Sciences Weihenstephan, Technical University of Munich, Freising, Germany; 3 Department of Chemistry, Aarhus University, Aarhus, Denmark; 4 Institute for Advanced Study, Technical University of Munich, Garching, Germany; 5 Department of Biochemistry and Molecular Pharmacology, New York University School of Medicine, New York, NY USA; 6 Present Address: Institut de Pharmacologie et de Biologie Structurale, Université de Toulouse, CNRS, UPS, Toulouse, France

**Keywords:** Plant molecular biology, Plant development

## Abstract

Auxins are plant hormones that direct the growth and development of organisms on the basis of environmental cues. Indole-3-acetic acid (IAA) is the most abundant auxin in most plants. A variety of membrane transport proteins work together to distribute auxins. These include the AUX/LAX protein family that mediate auxin import from the apoplast to the cytosol. Here we use structural and biophysical approaches combined with molecular dynamics to study transport by *Arabidopsis thaliana* LAX3, which is essential for plant root formation. Transport assays document high-affinity transport of IAA, as well as competitive behaviour of the synthetic phenoxyacetic acid auxin herbicide 2,4-dichlorophenoxyacetic acid and the auxin transport inhibitors 1-naphthoxyacetic acid and 2-naphthoxyacetic acid. Four cryo-EM structures were solved with resolutions of 2.9–3.4 Å: an inward open apo structure, two inward semi-occluded structures in complex with IAA and 2,4-dichlorophenoxyacetic acid, and a fully occluded structure in complex with 2-naphthoxyacetic acid. Structurally, LAX3 consists of a bundle and a scaffold domain. The ligand-binding site is sandwiched between these domains with two histidines occupying positions analogous to the sodium-binding sites in distantly related sodium:neurotransmitter transporters. This architecture suggests that these histidines couple transport to the proton motive force. Molecular dynamics simulations are used to explore substrate binding and release, including their dependence on specific protonation states. This study advances our understanding of auxin recognition and transport by AUX/LAX, providing insights into a fundamental aspect of plant physiology and development.

## Abstract (web summary)

This study reveals the structural basis of auxin import by the AUX/LAX family. LAX3 binds auxin as well as herbicides via a proton-coupled mechanism, which offers insights into hormone recognition that is essential for lateral root growth.

## Main

Auxins are a class of morphogenetic hormones that play a crucial role in growth and development in all plants^1^. Indole-3-acetic acid (IAA, p*K*_a_ = 4.7) is the quintessential auxin; it responds to complex environmental responses to coordinate growth patterns underlying various tropisms. In particular, IAA plays an essential role in signalling that directs plant shoots to grow towards the light (phototropism) and roots to penetrate the earth (gravitropism)^2^.

The physiological effects of auxin are implemented through a process termed polar auxin transport, in which a diverse group of transporters control the distribution of auxins throughout the various plant tissues^3^. An ion-trap mechanism based on the pH difference across the plasma membrane was initially proposed to govern cellular auxin import. In particular, protonated and thus neutral auxin molecules were hypothesized to diffuse passively into the cell, where the elevated pH causes deprotonation and the resulting negative charge traps auxins in the cytosol^4^. However, an Auxin Resistant1 (*aux1*) mutant was discovered with a severe agravitropic phenotype. This result, together with further work, indicated that IAA uptake is governed by the transport proteins from the Auxin Resistant1/Like-AUX1 (AUX/LAX) transporter family, with passive diffusion playing a minor role^5,6^.

**Extended Data Fig. 1:**
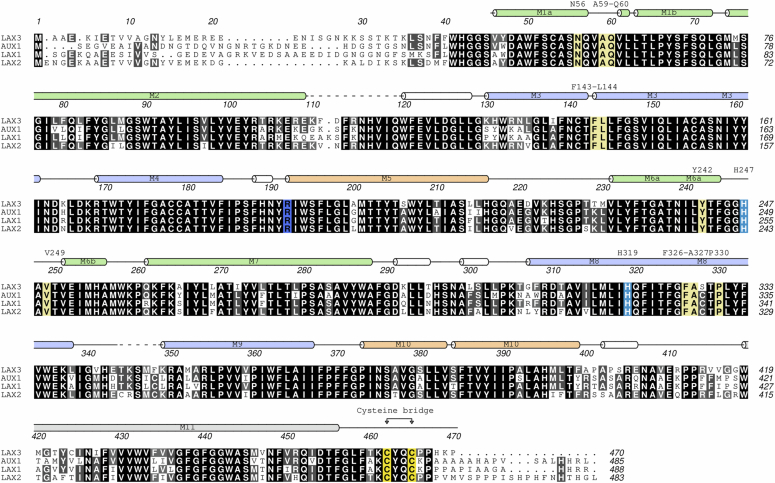
Multiple sequence alignment of LAX/AUX proteins found in *A. thaliana.* Alignment between *A. thaliana* LAX3 (Uniprot-ID: Q9CA25), LAX1 (Uniprot-ID: Q9LFB2), LAX2 (Uniprot-ID: Q9S836), and AUX1 (Uniprot-ID: Q96247). Conserved residues are highlighted in a grey scale. Coloured tubes represent α-helices found in the bundle domain (green), scaffold domain (light purple), arms (orange) and M11 (grey). Key residues are numbered above the α-helix markings and are highlighted in pale yellow. The C-terminally located disulfide bridge is highlighted in bright yellow.

In *Arabidopsis thaliana*, the AUX/LAX family comprises four members: AUX1 and LAX1–3, which all are homologous (74–82% sequence identity) but exhibit differential expression across tissues^7^ (Extended Data Fig. 1). AUX1 has been shown to be the main regulator of root gravitropism, while LAX3 is essential for lateral root development, vascular development and root hair formation^7,8^. Both AUX1 and LAX3 are required for phyllotaxis and apical hook formation^9,10^, whereas less is known about the physiological role of LAX1 and LAX2. In addition to physiological substrates, AUX/LAX proteins also transport synthetic auxin herbicides^5,6^. In particular, 2,4-dichlorophenoxyacetic acid (2,4-D, p*K*_a_ = 2.7) as well as possibly 1-naphthoxyacetic acid (1-NOA, p*K*_a_ = 4.2) and 2-naphthoxyacetic acid (2-NOA, p*K*_a_ = 3.2) compete with IAA and produce IAA-related phenotypes, suggesting that they also interact with AUX/LAX proteins^6,11,12^.

**Extended Data Fig. 2:**
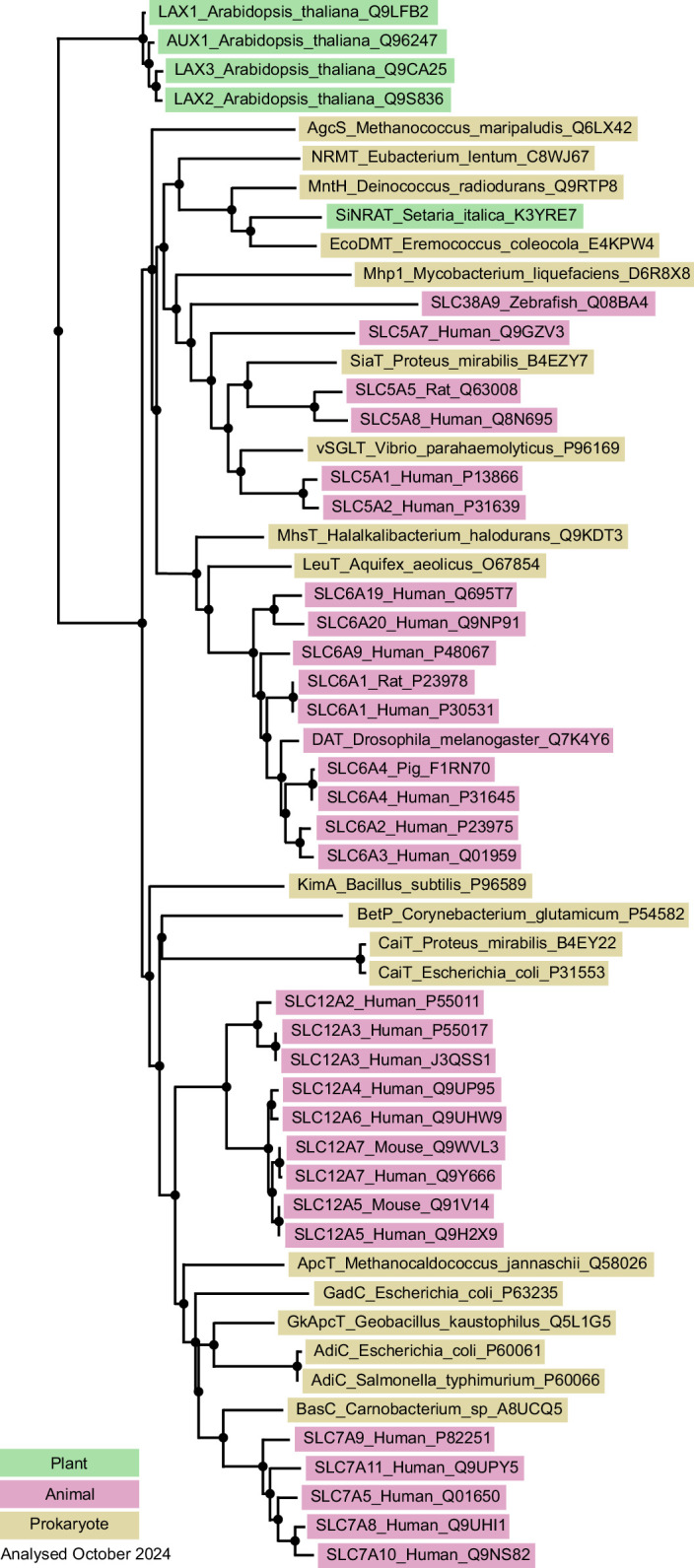
Phylogram of APC superfamily including the *Arabidopsis* LAX/AUX family and most solved APC structures. Phylogram of the relationship between *A. thaliana* AUX/LAX members and most solved APC structures (345 PDB codes from 50 unique protein sequences were identified in Oct 2024). AUX/LAX forms a distinct clade and share less 15% sequence identity to other structurally solved APC superfamily members.

Although AUX/LAX proteins are unique to plants, they belong to the ubiquitous auxin amino acid permease family, the largest clade within the amino acid–polyamine–organocation (APC) superfamily^13–15^. This superfamily includes LeuT, a well-studied bacterial amino-acid–sodium symporter, as well as members of the neurotransmitter–sodium symport (NSS) family responsible for neurotransmitter reuptake in animals (Extended Data Fig. 2). As implied by their name, a sodium gradient is used to drive transport by NSS members, and their structures show two well-defined sodium-binding sites (Na1 and Na2) on either side of the substrate-binding pocket^16,17^. These transporters adopt the APC fold (colloquially called the LeuT fold) consisting of ten transmembrane helices (M1–M10) divided into two pseudo-symmetric five-helix bundles. Structures of the inward and outward conformations imply a ‘rocking bundle’ mechanism, in which a mobile, bundle domain (M1–M2 and pseudosymmetric M6–M7) pivots relative to a static, scaffold domain (M3–M5 and M8–M10)^18–20^.

AUX/LAX transporters appear to utilize a proton gradient to drive the symport of auxin and protons, in contrast to the sodium coupling of well-characterized APC superfamily members such as LeuT and related NSS transporters^21^. AUX/LAX family members share less than 15% sequence identity with any APC transporter of known structure, and phylogenetic analysis supports the conclusion that they form a distinct clade (Extended Data Fig. 2). Therefore, questions about the molecular bases for substrate recognition, the proton–auxin stoichiometry and the energy coupling mechanism remain unresolved.

Here, we present a structural and biophysical characterization of *A. thaliana* LAX3. We determine four cryo-electron microscopy (cryo-EM) structures of LAX3 in three conformational states: a structure of an inward open apo state, two structures of an inward semi-occluded state in complex with IAA or 2,4-D and a structure of a fully occluded state with 2-NOA bound. These structural results are complemented with functional analysis of LAX3 activity and molecular dynamics (MD) simulations that address proton-dependent substrate release. Together, these data lead to a proposed transport mechanism and a ligand–inhibitor recognition scheme for the AUX/LAX family.

## Results

**Fig. 1:**
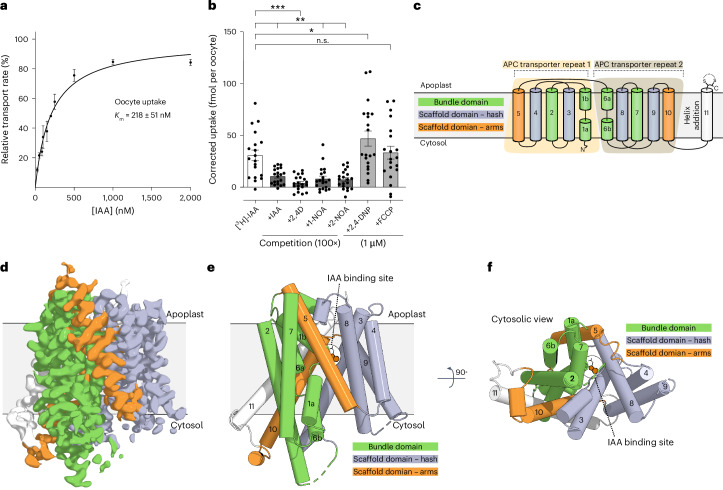
Activity and structural overview of LAX3. **a**, Kinetics of LAX3-mediated IAA uptake into *Xenopus* oocytes. Uptake can be described by a Michaelis–Menten model (*r*^2^ = 0.93, *K*_m_ = 218 ± 51 nM, *V*_max_ = 35 ± 19 fmol min^−1^ per oocyte). Data are presented as mean values ± s.e.m. Points represent rates derived from biologically independent time-course measurements, with raw data shown in Extended Data Fig. 3a (*n* = 5). **b**, The impact of inhibitors and competitors on LAX3-mediated IAA uptake into *Xenopus* oocytes at [^3^H-IAA] = 100 nM. Uptake of LAX3-injected oocytes in the presence of 10 µM IAA, 2,4-D, 1-NOA, 2-NOA or 1 µM of the proton decouplers 2,4-dinitrophenol (2,4-DNP) or carbonyl cyanide-p-trifluoromethoxyphenylhydrazone (FCCP). Each data point represents the uptake of one individual oocyte. The error bars show the mean ± s.e.m, *n* = 20 except [^3^H]-IAA where *n* = 19. One-way analysis of variance (ANOVA) followed by Dunnett’s multiple-comparisons test was used for data analysis. *P* values: [^3^H-IAA] versus [^3^H-IAA] + IAA: *P* = 0.0077, [^3^H-IAA] versus [^3^H-IAA] + 2,4-D: *P* = 0.0002, [^3^H-IAA] versus [^3^H-IAA] + 1-NOA: *P* = 0.0022, [^3^H-IAA] versus [^3^H-IAA] + 2-NOA: *P* = 0.0015, [^3^H-IAA] versus [^3^H-IAA] + 2,4-DNP: *P* = 0.0484, [^3^H-IAA] versus [^3^H-IAA] + FCCP: *P* = 0.9962. For uncorrected data, see Extended Data Fig. 3d. **c**, Topology diagram of LAX3 containing two APC transporter repeats (1 and 2), each comprising five transmembrane helices. The bundle domain (green) consisting of M1, M3, M6 and M8, and the scaffold domain (light purple) with M2, M4, M5, M7 and M9, form the core of the transporter. In addition, the transporter has two arms, M5 and M10 (orange), and an extra C-terminal helix, M11 (white), which breaks the internal symmetry of the transporter. **d**, The cryo-EM electrostatic potential map of the 3.21 Å structure of LAX3 bound to IAA, with colour coding as in **c**. **e**, A cartoon presentation of the LAX3 IAA-bound structure. The IAA-binding site is situated between the scaffold domain and the bundle domain and is highlighted with the chemical structure of IAA shown in orange. **f**, Top view of LAX3 seen from the cytosolic side, 90° rotation relative to **e**. The IAA binding site is highlighted as in **e**.

**Extended Data Fig. 3:**
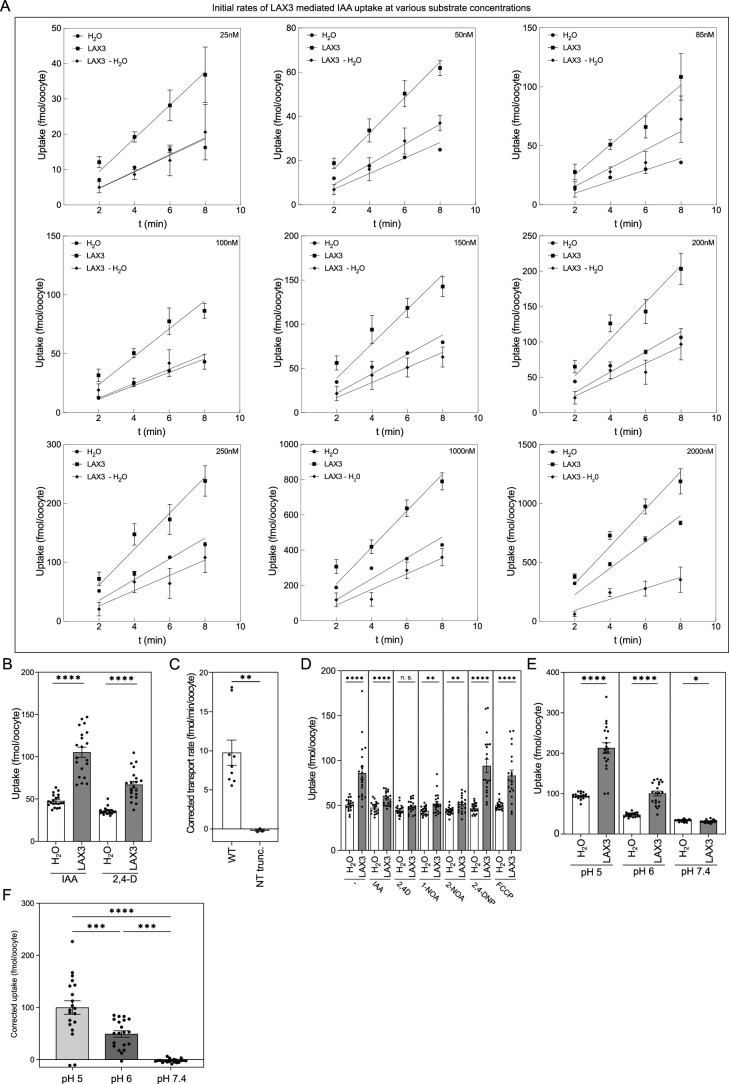
Supporting data panels for uptake assays conducted in *X. laevis.* **a**. Uncorrected uptake of IAA over time into *X. laevis* oocytes. Five individual oocytes were isolated at 2, 4, 6, and 8 min. The transport rate was determined using linear regression. To correct for passive uptake, the slope of water-injected oocytes was subtracted from LAX3-injected oocytes. Data points represent mean +/- SEM with *n* = 5. **b**. Radiolabeled IAA or radiolabeled 2,4-D uptake into *Xenopus* oocytes. Each data point represents the uptake of one individual oocyte. Data are presented as mean values +/- SEM with *n* = 20. Groups were compared by a two-sided unpaired Student’s t-test (IAA: P < 0.0001; 2,4-D: P < 0.0001). **c**. Impact of N-terminal truncation on LAX3-mediated IAA corrected uptake into *X. laevis* oocytes. Each data point represents the uptake of one individual oocyte. Data are presented as mean values +/- SEM with *n* = 9 (WT), *n* = 3 (NT trunc.). Groups were compared by a two-sided unpaired Student’s t-test (P = 0.0061). **d**. Uncorrected data from Fig. 1b. Impact of inhibitors, competitors and proton decouplers on LAX3-mediated IAA uptake into *X. laevis* oocytes. Uptake of LAX3 and H_2_O injected oocytes in the presence of 10 µM IAA, 2,4D, 1-NOA and 2-NOA or 1 µM of the proton decouplers 2,4-DNP and FCCP. Each data point represents the uptake of one individual oocyte. Data are presented as mean values +/- SEM with *n* = 20 (except FCCP, H_2_O with *n* = 18). Groups were compared by a two-sided unpaired Student’s t-test (“-“: P < 0.0001; IAA: P < 0.0001; 2,4D: P = 0.0703; 1-NOA: P = 0.0054; 2-NOA: P = 0.0022; 2,4-DNP: P < 0.0001; FCCP: P < 0.0001). **e**. Uncorrected IAA uptake of LAX3 and H2O injected oocytes at indicated pH. Each data point represents the uptake of one individual oocyte. Data are presented as mean values +/- SEM with *n* = 20 (except pH 5, H2O with *n* = 18 and pH 6, H2O with *n* = 19). Groups were compared by a two-sided unpaired Student’s t-test (pH 5: P < 0.0001; pH 6: P < 0.0001; pH 7.4: P = 0.0396). **f**. Corrected uptake of IAA mediated by LAX3. Each data point represents the uptake of one individual oocyte. Data are presented as mean values +/- SEM with *n* = 20. Groups were compared by a one-way ANOVA followed by Tukey’s multiple comparisons test (pH5 vs. pH6: P = 0.0002; pH5 vs. pH7.4: P < 0.0001; pH6 vs. pH7.4: P = 0.0002).

To characterize the function of AUX/LAX proteins, we expressed LAX3 from *A. thaliana* in *Xenopus* oocytes and monitored the uptake of radiolabelled IAA. The results show uptake of IAA (Extended Data Fig. 3a,b). Titration of this ligand demonstrates that LAX3 is a high-affinity transporter with an apparent *K*_m_ of 228 nM ± 51 nM (Fig. 1a). The properties of 2,4-D, 1-NOA and 2-NOA were also studied in competition assays with transport of IAA; these data demonstrate a competitive effect of all three compounds (Fig. 1b). Notably, proton uncouplers did not reduce transport (Fig. 1b). In addition, we used radiolabelled 2,4-D to show LAX3-mediated uptake of 2,4-D in oocytes (Extended Data Fig. 3b). LAX3 also exhibited pH-dependent transport of IAA, with the highest activity at pH 5.0 and an absence of uptake at pH 7.4 (Extended Data Fig. 3e,f).

**Extended Data Fig. 4:**
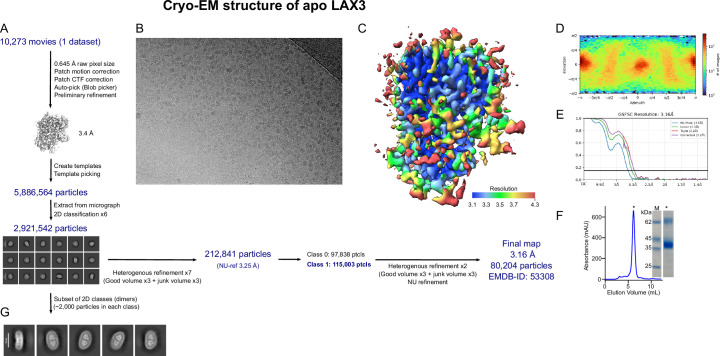
Image processing and map reconstruction of apo LAX3 in the inward open conformational state. **a**. Workflow of image processing and 3D reconstruction of apo LAX3. The processing was done in CryoSPARC v. 4.4.0. **b**. Representative micrograph showing the particle distribution of 10273 micrographs. **c**. Sharpened density map from the final non-uniform refinement colored by local resolution. **d**. Heat map showing the orientation distribution of the final particle stack. **e**. The corrected curve of the global Fourier shell correlation (FSC) indicates 3.16 Å based on the 0.143 gold-standard criterion. **f**. Representative size-exclusion chromatography profile and corresponding SDS-PAGE analysis of a LAX3 purification. Four individual purifications were carried out, one for each cryo-EM structure. **g**. Dimeric LAX3 2D-classes from a small subset of the particles.

**Extended Data Fig. 5:**
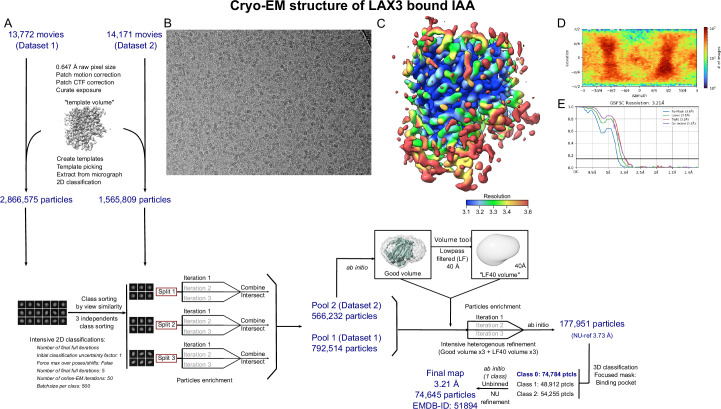
Image processing and map reconstruction of IAA bound LAX3 in the semi-occluded conformational state. **a**. Workflow of image processing and 3D reconstruction of LAX3 with IAA. The processing was done in CryoSPARC v. 4.4.0. **b**. Representative micrograph showing the particle distribution of 27943 micrographs. **c**. Sharpened density map from the final non-uniform refinement colored by local resolution. **d**. Heat map showing the orientation distribution of the final particle stack. **e**. The corrected curve of the global Fourier shell correlation (FSC) indicates 3.21 Å based on the 0.143 gold-standard criterion.

**Extended Data Fig. 6:**
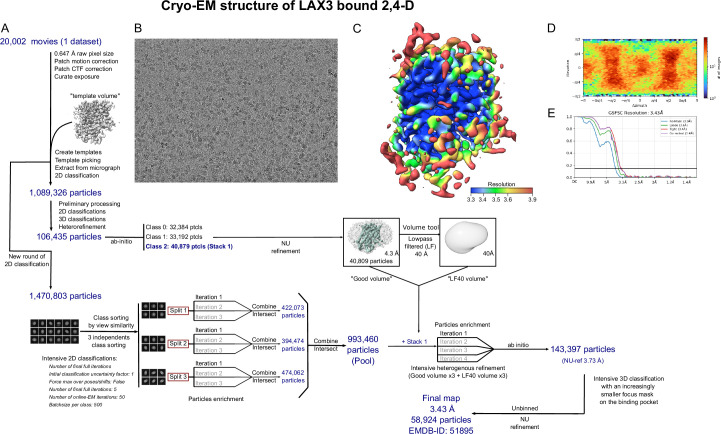
Image processing and map reconstruction of 2,4-D bound LAX3 in the semi-occluded conformational state. **a**. Workflow of image processing and 3D reconstruction of LAX3 with 2,4-D. The processing was done in cryoSPARC v. 4.4.0. **b**. Representative micrograph showing the particle distribution of 20002 micrographs. **c**. Sharpened density map from the final non-uniform refinement colored by local resolution. **d**. Heat map showing the orientation distribution of the final particle stack. **e**. The corrected curve of the global Fourier shell correlation (FSC) indicates 3.43 Å based on the 0.143 gold-standard criteria.

**Extended Data Fig. 7:**
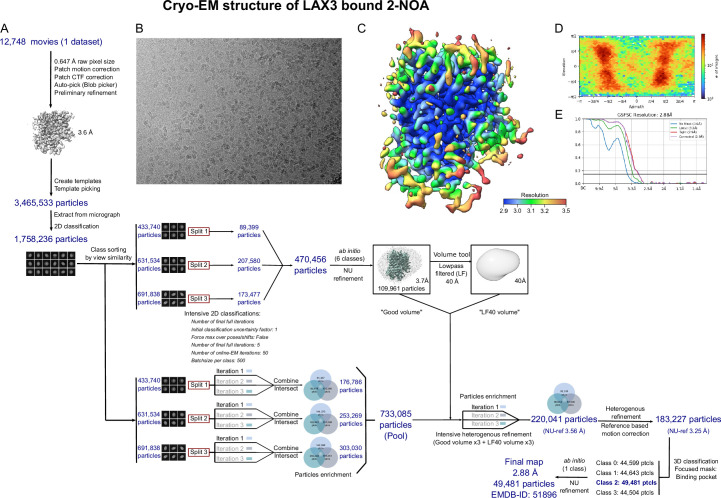
Image processing and map reconstruction of 2-NOA bound LAX3 in the occluded conformational state. **a**. Workflow of image processing and 3D reconstruction of LAX3 with 2-NOA. The processing was done in cryoSPARC v. 4.4.0. **b**. Representative micrograph showing the particle distribution of 12748 micrographs. **c**. Sharpened density map from the final non-uniform refinement colored by local resolution. **d**. Heat map showing the orientation distribution of the final particle stack. **e**. The corrected curve of the global Fourier shell correlation (FSC) indicates 2.88 Å based on the 0.143 gold-standard criterion.

For structural studies, different constructs were screened and expressed in *Saccharomyces cerevisiae*. Based on the yield and stability of the purified proteins, an N-terminally truncated construct of *A. thaliana* LAX3 (residues 44–470) was used for single-particle cryo-EM without a ligand (apo state) or in the presence of either IAA, 2,4-D or 2-NOA (Extended Data Figs. 1 and 4–7). Transport assays with the truncated construct indicate that removal of the N terminus prevents activity in oocytes (Extended Data Fig. 3c); nevertheless, the resulting four structures revealed ligand-bound states with a well-defined binding pocket (Extended Data Figs. 4–7 and Extended Data Table 1). In other APC superfamily members, the N terminal has been known to be involved in regulation (for example, ref. ^22^). While our oocyte uptake assays support the importance of the N terminal for the transport activity of LAX3, they do not invalidate the observed substrate-binding poses or the overall architecture of the protein, which are clearly defined by the map density and correlate well with other structures in similar conformational states in the APC superfamily. The expected 11 transmembrane helices are well resolved in all four maps; however, flexible regions including two short loops (residues 110–114 and 342–347) and the last few residues on the C terminus (residues 466–470) could not be modelled.

The LAX3 monomer consists of 11 transmembrane helices (M1–M11) (Extended Data Fig. 4). The first ten helices adopt the canonical APC fold with two repeats inverted relative to the plane of the membrane (Fig. 1c–f). These are followed by M11, which contains a disulfide bridge on the apoplastic side of the membrane (Cys462–Cys465) near the C terminus (Fig. 1c and Extended Data Fig. 1). Each of the two pseudo-symmetric repeats (M1–M5 and M6–M10) contributes to two distinct domains that correspond to the bundle domain and the scaffold domain, as previously defined for the APC fold. The bundle domain contains the first two helices of each repeat (M1–M2 and M6–M7), where the first helix (M1 and M6) is unwound and bent in the middle of the membrane. The scaffold domain contains the last three helices (M3–M5 and M8–M10) and is often divided into a ‘hash’ (M3–M4 and M8–M9) and arms (M5 and M10)^23^. Ligand binding takes place within a central binding pocket at the interface between the bundle and scaffold domain (Fig. 1e,f).

**Fig. 2:**
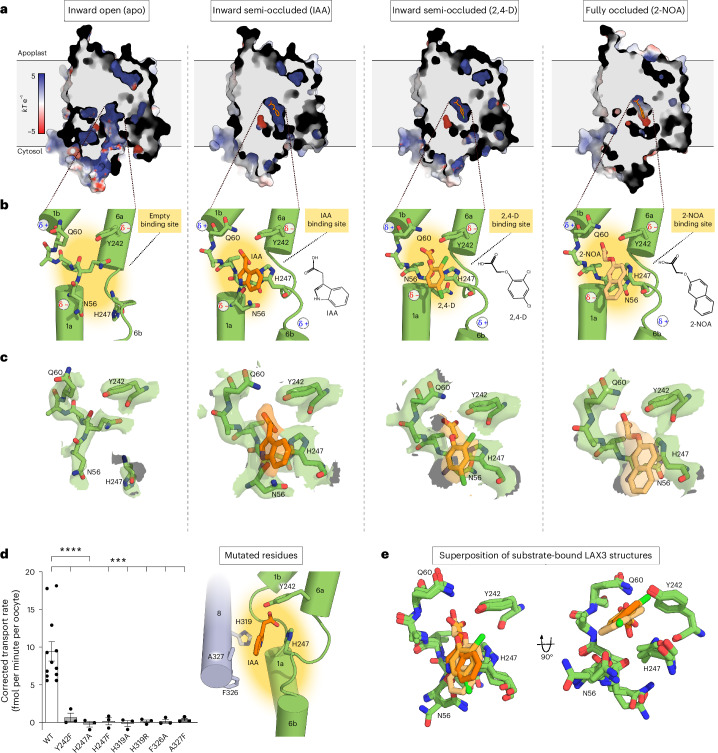
The LAX3 binding site. **a**, A cut-through view of the surface presentation of LAX3 structures coloured by electrostatic potential (*kT* e^−1^). From left to right: inward open, LAX3 apo; inward semi-occluded, LAX3 IAA and LAX3 2,4-D; and fully occluded, LAX3 2-NOA. **b**, A close-up view of the binding site. From left to right: inward open LAX3 apo; inward semi-occluded, IAA and 2,4-D; and fully occluded, 2-NOA. The dipole moments of surrounding helices are shown as well as a chemical presentation of the individual ligands. **c**, Density in the binding site surrounding the substrates (orange) and interacting residues (green). From left to right: inward open LAX3 apo; inward semi-occluded IAA and 2,4-D; and fully occluded 2-NOA. The map surface represents the density level at 10 standard deviations (σ) above the mean. **d**, Left: uptake of IAA by LAX3 point mutants into *Xenopus* oocytes at [^3^H-IAA] = 100 nM. Data are presented as mean values ± s.e.m. with *n* = 3 except WT where *n* = 12. Data analysis was performed by one-way ANOVA followed by Dunnett’s multiple-comparisons test. Right: structural overview of mutated residues from the bundle domain (green) and the scaffold domain (light purple). *P* values: WT versus Y242F: *P* = 0.0004, WT versus H247A: *P* < 0.0001, WT versus H247F: *P* = 0.0002, WT versus H319A: *P* = 0.0001, WT versus H319R: *P* = 0.0002, WT versus F326A: *P* = 0.0002, WT versus A327F: *P* = 0.0002. **e**, Superposition of the inward semi-occluded (IAA and 2,4-D) and fully occluded (2-NOA) LAX3 structures.

In the absence of ligands, LAX3 adopts an inward open conformational state in which the central binding pocket is empty. Access to the binding pocket from the apoplastic side of the membrane is completely blocked by a thick gate formed from M1b and the loop between M7 and M8 and is fully accessible from the cytosolic side (Fig. 2a–c).

In the presence of IAA, well-defined density is seen in the binding pocket (Fig. 2c). In this IAA-bound state, access from the cytosol is constricted by a thin gate composed of His189 and Arg192 on the M4–M5 loop, Val249 and Glu252 on M6b, and Phe326 and Thr329 on M8. This architecture defines the IAA-bound structure as an inward semi-occluded bound conformational state in which the ligand binding site is still water accessible (Fig. 2a).

**Extended Data Fig. 8:**
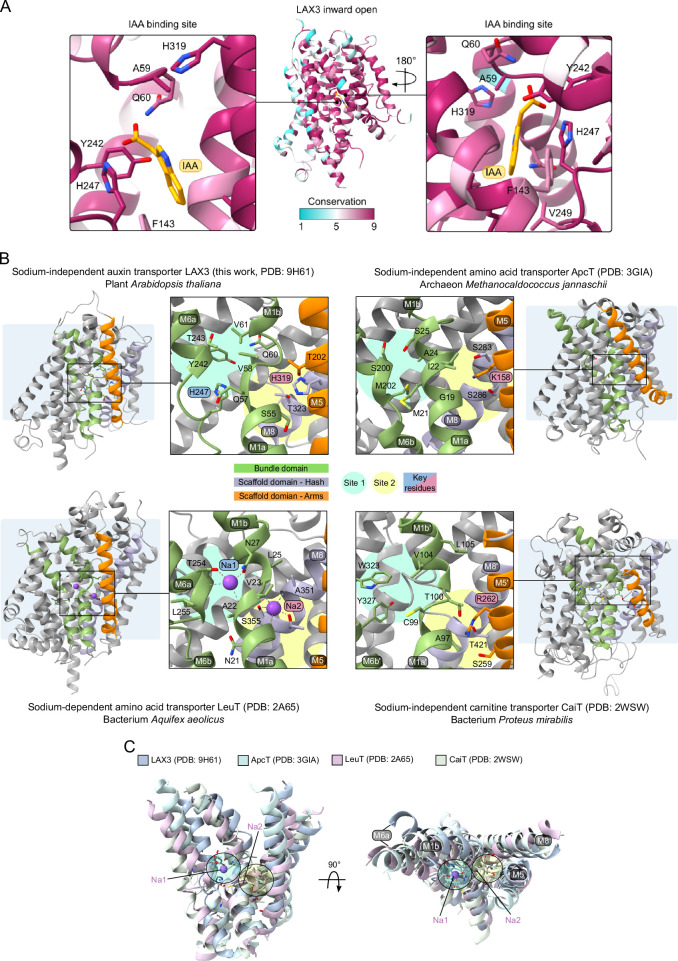
Conservation of residues among LAX3 proteins and comparison of sodium site 1 and site 2 LeuT to similar position in LAX3 and other APC proteins. **a**. The model of LAX3 (cartoons) bound to IAA (orange) was submitted to ConSurf and colored according to the degree of residue conservation as blue (least conserved), white (moderately conserved) or maroon (highly conserved). Two close-up views of the amino acids in the binding pocket as sticks show that they are highly conserved. **b**. Structures of sodium-independent transporters of auxin (LAX), amino acids (ApcT) and carnitine (CaiT) are compared with a sodium-dependent transporter of amino acids (LeuT). Models are shown as grey cartoons while helices that contribute residues to site 1 or site 2 are colored green (M1 and M6 of the bundle domain), orange (M5 of the scaffold domain) and light purple (M8 scaffold domain). The location of site 1 and site 2 are shown in cyan and yellow circles, respectively. Key residues involved in charge stabilization of the sodium-independent transporters are shown in blue or red, while the two sodium ions that fulfill the same role in the sodium-dependent LeuT protein are also shown in blue and red. **c**. Superposition of helices M1, M5, M6 and M8 of LAX3 (dark blue), ApcT (light blue), LeuT (pink) and CaiT (light green). For both the side and top view, the location of site 1 and site 2 are shown with a cyan and yellow circle, respectively, as well as the sodium ions coordinated by LeuT.

The binding pocket can be roughly divided into a polar and a hydrophobic region, with the polar region being above the ligand (apoplast oriented) and the hydrophobic region being below the ligand (cytosol oriented). The polar region is defined by His247 together with the negative dipole of M6a and other polar moieties from the unwound region on M1. IAA is bound with its carboxylate group in this polar region pointing towards the apoplast side and interacting with the unwound portions of M1 and M6. The carboxylate interacts with Gln60(M1), Tyr242(M6), His247(M6) and the backbone amide of Ala59(M1). This network of interactions is further stabilized by the unwound part of M1 that holds Gln60 in place (Fig. 2b). Abolishing any of these interactions by mutagenesis, such as Y242F, results in loss of transport (Fig. 2d). The indole ring of IAA is situated in the hydrophobic region defined by Phe143(M3), Leu144(M3), Val249(M6), Phe326(M8), Ala327(M8) and Pro330(M8). Here, the indole ring occupies the space between M1 and scaffold helices M3 and M8, with hydrogen bonding to the side-chain amide and main-chain carbonyl of Asn56(M1). In addition, hydrophobic interactions are formed between the indole ring and the M8. Changing the shape of the binding pocket by introducing either A327F or F326A results in loss of transport (Fig. 2d). The extensive interactions of the binding pocket to the substrate show that shape complementarity plays a major role in substrate selection by LAX3. All residues defining the binding pocket show very high sequence conservation across different plant species and are fully conserved in all *A. thaliana* AUX/LAX proteins (Extended Data Figs. 1 and 8a).

In sodium-driven APC transporters, two sodium-binding sites are located next to the substrate-binding pocket and are essential for transport. In LAX3 and other AUX/LAX proteins, two fully conserved histidines, His247(M6) and His319(M8), occupy similar positions as the sodium ions. These histidines represent potential proton donor and acceptor sites for coupling transport of auxin to the proton motive force^24^. As they are the only protonatable residues in the vicinity of the binding pocket (Fig. 2b and Extended Data Fig. 1), we addressed their impact on transport using point mutations expressed in *Xenopus* oocytes (Fig. 2d). We found that substitution of either histidine with alanine (H247A and H319A), arginine (H319R) or phenylalanine (H247F) rendered LAX3 inactive, supporting their central role in transport. His247 is of particular interest because it engages directly with the carboxyl group of IAA.

The 2,4-D-bound structure of LAX3 adopts a similar inward semi-occluded conformational state as the IAA-bound structure with root mean square deviation of Cα (r.m.s.d._Cα_) of 0.5 Å (Fig. 2a). There is clear density for the phenoxy ring system and carboxylate of 2,4-D, while the density for the aryl oxygen is weaker. This is sufficient to confirm a pose for this synthetic auxin substrate similar to that of IAA (Fig. 2c). The interaction network coordinating 2,4-D mimics the one observed for IAA, involving the same residues on M1 and M6 (Fig. 2b) and with the phenoxy ring occupying the same hydrophobic region of the binding pocket. As the phenoxy ring lacks the nitrogen found in the indole ring of IAA, it prevents Asn56(M1) from interacting.

**Fig. 3:**
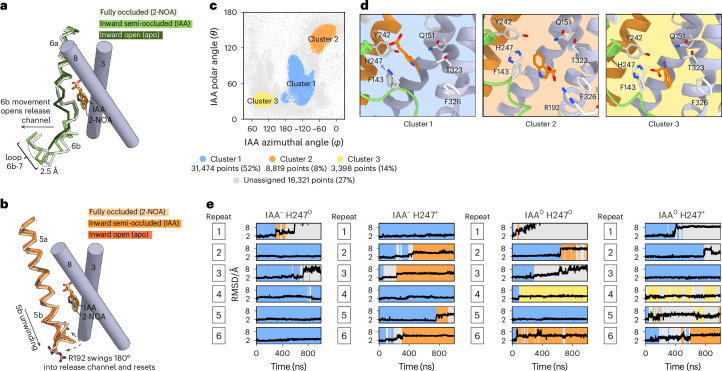
Dynamics of LAX3. **a**, A cartoon representation highlighting movement in M6b and the M6b-7 loop of the bundle domain, as seen when going from the fully occluded conformational state (LAX3 2-NOA) to the inward semi-occluded conformational state (LAX3 IAA) and the inward open conformational state (LAX3 apo). **b**, Changes observed in the arms region of LAX3, including unwinding of M5b, when transitioning from the fully occluded state (2-NOA) to the inward semi-occluded state (IAA). For both **a** and **b**, M3 and M8 of the scaffold domain is shown in grey. **c**, The polar (*θ*) and unwrapped azimuthal (*φ*) angle (addressed in Extended Data Fig. 9) of IAA within the binding site used for clustering of the ligand into three distinct orientation clusters (cluster 1 in blue, cluster 2 in orange and cluster 3 in yellow). The remaining data points (grey) were unassigned. **d**, A close-up view of the representative (centroid) IAA poses for each of the three clusters assigned in **c**. **e**, The r.m.s.d. of IAA, with the background colour indicating the assigned cluster group for each simulation frame.

In contrast to the other structures, the 2-NOA-bound form of LAX3 adopts a fully occluded conformational state (Fig. 2a). The scaffold domain is unchanged relative to the inward semi-occluded state (r.m.s.d._Cα_ = 0.4 Å), but the bundle domain has rotated approximately 10°. As a result, the M6b helix moves closer to M4–M5 helices and the scaffold domain, which narrows the cytosolic exit path by ~2.5 Å and results in a full occlusion of the binding pocket (Figs. 2a and 3a). Concurrent with this rigid body movement, the cytosolic end of M5 is extended by one turn, causing Arg192 to swing 180° away from the binding pocket (Fig. 3b). The density for 2-NOA is well defined and illustrates a different interaction pattern relative to IAA and 2,4-D (Fig. 2c). As with IAA and 2,4-D, the carboxylate of 2-NOA is in hydrogen bonding distance to Tyr242 and the unwound region of M1 (Fig. 2b). However, the carboxylate has lost interaction with His247. Instead, the aryl oxygen of 2-NOA generates novel interactions with Tyr242, Gln151 and His247, pushing the carboxylate group up towards the apoplast relative to IAA. Given the key location of His247 at the junction in the binding pocket, analogous to the Na1 site in sodium-driven APC transporters, this change in interaction with His247 appears to be a key aspect of 2-NOA binding and possibly the cause of its inhibitory action (Fig. 2b).

**Extended Data Fig. 9:**
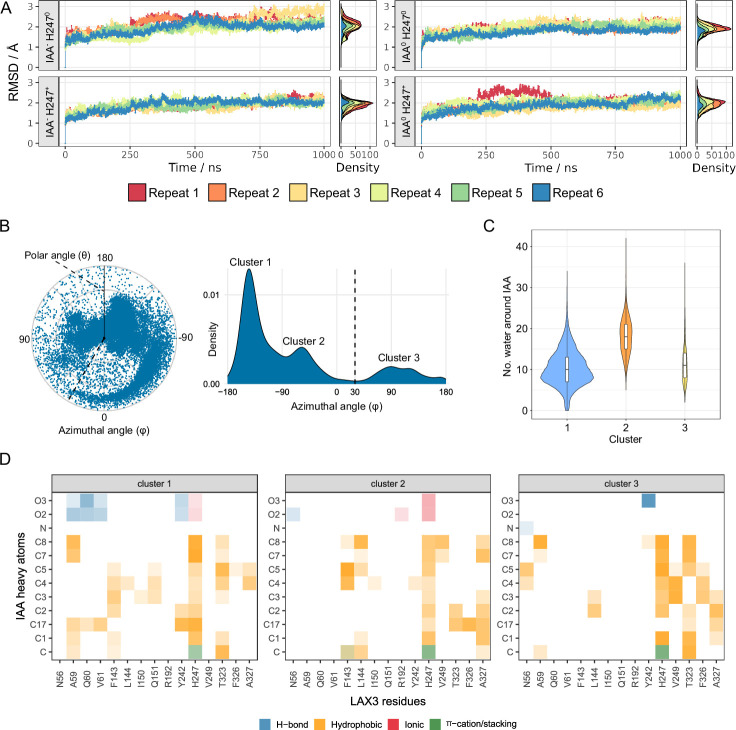
Molecular dynamics simulation of IAA in the binding site. **a**. Cα RMSD values describing the movement of the LAX3 protein in different protonation scenarios over 1 µs of simulations and with six repeats for each scenario. The stacked density plots indicate the distribution of RMSD values for each simulation trajectory. **b**. The spherical angles of IAA within the binding site shown in a circular plot, with a wrapped azimuthal (φ) angle [-180°,180°] mapped on the edge of the circle, and a linear polar (θ) angle [0,180°] mapped on the radial line. The density plot of the azimuthal (φ) angle values shows the least populated angle value (30°), which was used as a plot border in the unwrapped dataset plot (Fig. 3a). **c**. Water count around IAA grouped by the assigned cluster as shown in B and Fig. 3. Box plot elements represent the following: centre line: median (50th percentile), box minimum: 25th percentile (Q1), box maximum: 75th percentile (Q3), box height: interquartile range (IQR) (between 25 and 75%), whisker lower edge: Q1 - 1.5 x IQR, whisker upper edge: Q2 + 1.5 x IQR. **d**. ProLIF fingerprint analysis showing residues in LAX3 that interact with IAA in simulations, divided by cluster. Colors indicate the interaction type (blue for H-bond, yellow for hydrophobic, red for salt-bridges and green for pi-stacking). The color intensity corresponds to the normalized frequency of the interactions in each cluster (where the 100% intensity was assigned to the most frequent protein-IAA interaction in each cluster).

To explore the role of His247 in proton coupling as well as the dynamics of ligand binding, we performed MD simulations of LAX3 in a membrane bilayer with IAA in the binding site. Six independent simulations were run for each of four possible proton configurations: IAA either protonated (IAA°) or negatively charged (IAA^−^), and His247 either protonated (H247^+^) or neutral (H247°). These states correspond to the net transport of zero protons (IAA^−^/H247°), one proton (IAA°/H247° and IAA^−^/H247^+^) or two protons (IAA^0^/H247^+^). Each run had a duration of 1 µs, with a total of 6 μs for each configuration. Although there were no large-scale conformational changes, we observed notable changes in atomic interactions and the ligand pose of IAA within the binding site (Extended Data Fig. 9a,b). Cluster analysis of the simulations reveals three distinct poses adopted by IAA (Fig. 3c,d). Cluster 1 is the largest and corresponds to variations of the initial pose taken from the cryo-EM structure. In cluster 2, the ligand is flipped 180°, whereas in cluster 3 the carbonyl is kinked and rotating freely. The flipped cluster 2 configuration is associated with an increased hydration of the binding pocket, where the number of water molecules surrounding the ligand increases from ~10 to ~18 (Extended Data Fig. 9c). The flipped pose of IAA position the IAA carbonyl in the vicinity of Arg192, which serve as a coordination partner and a mechanism for leading the ligand out of the binding pocket towards the cytosol. In one replicate of IAA^−^/H247^0^ (repeat 1), we observed IAA flipping within the binding site to establish an interaction with Arg192, followed by IAA exiting the binding site while retaining the charge–charge interaction with Arg192 (Extended Data Video 1). This behaviour also lends meaning to the movement of the M6b helix and the 180° swing of Arg192 in the transition from the fully occluded state to the inward semi-occluded state seen in the cryo-EM structures (Fig. 3a,b).

We propose that cluster 2 is consistent with ligand solvation and its initial release from the pocket. In the IAA^−^/H247^+^ simulations, this flipped pose displays a rather consistent r.m.s.d., indicating a low degree of fluctuations and, thus, a metastable binding configuration. By contrast, the relatively high fluctuations in other simulation systems are indicative of incorrect protonation patterns (Fig. 3e). This is further supported by protein–ligand interaction analysis (fingerprinting), where cluster 3 has markedly lower frequency of hydrogen bonds and an abundance of low-frequency hydrophobic interactions, as a result of non-specific binding (Extended Data Fig. 9d). The MD runs are supportive of a model where one proton is carried by the His247–auxin couplet. It remains to be tested whether His319 might carry an additional proton in certain physiological conditions.

## Discussion

Here, structures of LAX3 have been experimentally determined both in an apo state and bound to IAA, 2,4-D and 2-NOA. The structures portray three distinct key conformations from the transport cycle. Combined, the structures show that LAX3 adopts the APC fold and reveals the binding pose and coordination scheme of three relevant compounds, which provide insight into the dynamics that generally control substrate binding. The in vivo transport assays highlight the importance of key residues in the central binding pocket. MD simulations explore the role of protonation in ligand binding and reveal a plausible mechanism for substrate release. In addition, the MD results are highly complementary to the EM structural data, where similar regions of mobility are observed, exemplified by the movements of M5b and Arg192 (Fig. 3b and Extended Data Video 1).

The two conserved histidine residues on either side of the ligand are found not only in *A. thaliana* AUX/LAX members but also in all AUX/LAX members across other plant species, supporting their relevance to the transport process (Extended Data Fig. 8a). These histidines reside at positions that are equivalent to the sodium-ion positions in the related transporters from the APC superfamily (Extended Data Fig. 8b,c). The interactions between sodium ions and the two APC sodium-binding sites have been a major focus in the past and energetically couple substrate transport to a sodium gradient^16,17^. The protonation state of the histidines in LAX3 is thus likely to play a similar pivotal role in coupling auxin transport to the proton motive force to energize transport. This proposed pivotal role of the two histidines is indicative of a general AUX/LAX mechanism of transport. However, this might not be conserved among all sodium-independent APC protein families, as some distantly related families (<10% sequence identity) do not have these central histidines^25,26^.

We suggest a proton-coupled mechanism for LAX3 with a stoichiometry of one proton per IAA molecule. At the low pH of the apoplast (pH 5.5), ~14% of IAA molecules (p*K*_a_ = 4.7) are protonated, and assuming a standard p*K*_a_ for histidine (6.5), ~75% of His247 residues would be protonated. Thus, protons are likely to be driven into the active site in the outward conformation. This proton would ultimately be shared in a hydrogen bond between auxin and His247, creating a joint donor–acceptor region formed between the carboxylate and the side chain of His247.

MD simulations illustrate the dynamics of IAA within the binding site in the inward semi-occluded state. Notably, they show that IAA can flip 180° and interact with Arg192, an interaction that requires dynamic changes to the M5 helix. Indeed, cryo-EM structures show unwinding of the M5 helix that swing Arg192 into the binding pocket as it loosens and becomes increasingly hydrated (Fig. 3b and Extended Data Video 1). Taken together, these results suggest that Arg192 guides the deprotonated IAA out of its binding pocket and into the cytosol. Assuming that the higher pH would induce release of the proton from His247, this process would result in an electroneutral, 1:1 stoichiometry of H^+^:IAA^−^. Although previous studies have not explicitly addressed this stoichiometry, auxin transport has been reported to be correlated to an overall dissipation of cellular membrane potential in vivo, implying that either the process itself or some downstream response to auxin is electrogenic^21,27–29^. It is known that auxin transport is linked to increased proton pumping by the proton P-type ATPase, which could explain this observation, but it is also possible that His319, which occupies the Na1 site in related Na-dependent APC transporters, could be recruited to bind another proton under certain conditions to create electrogenic transport^30,31^. In addition, we have tried to measure electrogenic transport of full-length LAX3 using solid-supported membrane electrophysiology, but the LAX3 proteoliposomes do not display electrogenic behaviour in our hands. While this could be due to issues with sample preparation or experimental setup, this could also be interpreted as further support that LAX3 can mediate electroneutral transport.

**Fig. 4:**
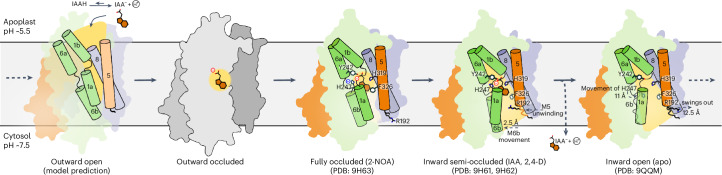
Proposed mechanism for auxin import by LAX3. In the outward open conformation, IAA enters the binding site with a proton. In the occluded conformations, IAA is stabilized in the central binding pocket by its interaction to His247 and surrounding residues. On the cytosolic side, IAA and the proton are then released, involving unwinding of M5 and movement of Arg192.

We propose a transport model that follows the rocking bundle mechanism (Fig. 4). According to this mechanism, the bundle domain starts in an outward open position. IAA then binds either in its protonated state or in conjunction with a proton that is shared with His247, stabilized by the negative dipol of M6a. The consequent coordination of residues at the top of the binding pocket drives a conformational transition through the occluded state, involving a rocking movement of the bundle domain into the inward state. The inward conformation promotes hydration of the binding pocket and extension of the cytosolic end of the M5 helix with a swing of Arg192 to the cytosolic side of the binding pocket. This positive charge stabilizes the charged IAA^−^ molecule as it flips in the binding site and readies for release to the cytosol. A similar flipping of auxin in the transition between inward and outward states has previously been documented in PIN-FORMED auxin exporter proteins^32–34^. Some APC proteins display transient unwinding of the M5 helix (‘the arm’), which has been associated with release of sodium from the Na2 site (equivalent to His319) and progression from inward occluded to inward open conformations^35,36^. It is thus notable that LAX3 displays a similar behaviour that is linked to the 180° flip of Arg192 (Fig. 3b). Although the specifics of conformational change in M5 are different, the role of dynamics in this structural element may be a recurring theme in the transport mechanism of the APC superfamily.

In summary, we have presented structural, biophysical and computational analyses of LAX3 leading to a mechanistic model elucidating how AUX/LAX proteins bind and transport substrates. Structures confirm that three different ligands, IAA, 2,4-D and 2-NOA, all occupy the same binding pocket in LAX3. The structures reveal distinct interactions that explain substrate recognition and could be targeted for future modification and modulation of hormone substrate recognition. This work provides a comprehensive foundation for future studies aiming to elucidate AUX/LAX function in the context of plant growth and development.

## Methods

### Protein purification

*A. thaliana* LAX3 (UniProt accession code Q9CA25) was cloned in a yeast expression construct based on p423_GAL1 in the frame with a C-terminal tobacco etch virus (TEV) protease cleavage site and a deca-histidine purification tag. The *S. cerevisiae* strain DSY-5 with transformed plasmid was cultivated in 5-l shaking flasks to high cell density and collected after 22-h induction with galactose or in a bioreactor^37^. Cell pellets were washed three times in water and resuspended in buffer A (0.1 M Tris pH 7.5, 0.6 M NaCl and 1.2 mM phenylmethylsulphonyl fluoride). After cell disruption, by bead beating, membrane fractions were sedimented by ultracentrifugation at 200,000*g* for 2 h and resuspended in buffer B (0.05 M Tris pH 7.5, 0.5 M NaCl and 20% glycerol), aliquoted and stored at −80 °C.

The cell membrane was thawed and solubilized in buffer C (0.05 M Tris pH 7.5, 0.5 M NaCl and 10% glycerol) with 1% *n*-dodecyl-β-d-maltoside (DDM) and 0.1% cholesterol hemisuccinate (CHS) under agitation at 4 °C. Insoluble material was removed by filtering the sample through a 5-µm and 1.2-µm filter. The supernatant was supplemented with 20 mM of imidazole (pH 7.5) and subsequently loaded on a 1-ml nickel–nitrilotriacetic (Ni–NTA) column. The column was washed with 20 column volumes (CV) of buffer D (buffer A containing 20 mM imidazole, 0.1% DDM and 0.01% CHS) and 20 CV buffer E (buffer A containing 70 mM imidazole, 0.026% DDM and 0.0026% CHS). Next, the column was washed with 20 CV of buffer F (0.05 M 4-(2-hydroxyethyl)-1-piperazineethanesulfonic acid (HEPES) pH 7.5 0.15 M NaCl, 10% glycerol, 0.0087% DDM and 0.00087% CHS) and then 20 CV with buffer G (0.05 M HEPES pH 7.5, 0.15 M NaCl, 10% glycerol, 0.05% (50xCMC) lauryl maltose neopentyl glycol and 0.005% CHS). After the detergent exchange, the column was washed again with 10 CV buffer H (0.05 M HEPES pH 7.5, 0.15 M NaCl, 10% glycerol, 0.003% (3xCMC) lauryl maltose neopentyl glycol (LMNG) and 0.0003% CHS) and eluted with buffer H supplemented with 500 mM imidazole. The TEV protease was added to the eluate and dialysed overnight against buffer F supplemented with 0.5 mM tris(2-carboxyethyl)phosphine. The sample was then filtered and rerun on a Ni–NTA column to adsorb the His-tagged proteins consisting of TEV protease, cleaved tag and uncleaved tagged protein. The flow-through fraction, containing tag-free LAX3, was concentrated on a 50 kDa cut-off centricon (Vivaspin) and polished by size-exclusion chromatography on a Superdex200 10/300 column. The protein samples that were used for the cryo-EM samples resulting in the LAX3 bound to IAA, 2,4-D or 2-NOA were run in gel filtration buffer I (0.05 M HEPES pH 7.5, 0.15 M NaCl, 0.001% LMNG and 0.0001% CHS), while the sample that was used for the apo LAX3 structure was run in gel filtration buffer II (0.05 M 2-morpholinoethanesulphonic acid pH 5.5, 0.15 M NaCl, 0.001% LMNG and 0.0001% CHS). Protein was concentrated using Amicon Ultra Centrifugal Filter (50 kDa molecular weight cut-off).

### Cryo-EM sample preparation

The Quantifoil R-1.2/1.3, Cu-300 mesh grids were glow-discharged for 45 s at 15 mA in a GloQube Plus (Quorum) for the preparation of cryo-EM. Before the blotting session, LAX3 (8 mg ml^−1^) was incubated with the following concentrations of IAA (5 mM) or 2,4-D (5 mM) or 2-NOA (5 mM) for 30 min, or the apo LAX3 was blotted without any substrate. A drop of 4 µl of the LAX3 sample was applied to the glow-discharged grids, blotted with a Vitrobot Mark IV (ThermoFisher Scientific) using the following settings (temperature 4 °C, 100% humidity, blot time of 4 s, blot force −1) and vitrified in liquid ethane.

### Image collection and data processing

Two different Titan Krios G3i microscopes (ThermoFisher Scientific) operating at 300 kV and equipped with a BioQuantum Imaging Filter (energy slit width of 20 eV) with a K3 detector (Gatan) were used to collect the movies. The LAX3 in complex with IAA, 2,4-D and 2NOA datasets were collected on Krios II at EMBION (the Danish National Cryo-EM Dacility) while the apo LAX3 dataset was collected on Krios I at eBIC (the UK’s National Cryo-EM Facility). The datasets were acquired using automated acquisition EPU Software at a nominal 130,000 magnification corresponding to a physical pixel size of 0.647 Å (EMBION) or 0.645 Å (eBIC). For all datasets, the movies were saved in super-resolution pixel size and binned 2× in EPU Software back to the nominal pixel size.

Gain-normalized micrographs were imported into cryoSPARC^38^ and processed for patch motion correction, patch contrast transfer function estimation and particle picking with a blob picker 27. The majority of the LAX3 particles appeared monomeric, while a subpopulation (too small for reconstruction) exhibited dimeric behaviour (Extended Data Fig. 4g). After several rounds of particle cleaning, an initial preliminary volume map was used to create templates for template picking. From a full dataset of 2-NOA LAX3 with 12,748 movies, template picking provided a total of 3,465,533 particles. After rounds of two-dimensional (2D) classifications, manually selected 2D classes yielded a total of 1,758,236 particles (pool 1), which were reextracted with a box size of 384 pixels (Fourier crop to box size 192). Processing of the EM data was challenging, owing to the lack of fiducial markers and the limited size of the protein (47 kDa) as well as the presence of empty detergent micelles of similar size. First, the starting pool 1 particles were split into three stacks based on their 2D class resemblance: split 1-top/bottom view, split 2-angle view and split 3-side view. Each split went through an iterative 2D classification with indicated parameters in which only a small portion of the most dissimilar 2D classes were eliminated. The 2D classification was stopped when the percentage of the selected good 2D classes represented more than 95% of the particles from the previous round. The good particle stacks from each individual 2D classification were recombined, resulting in a total of 470,456 particles. Following an ab-initio model reconstruction, particles from the good class were used for non-uniform refinement with C1 symmetry imposed, resulting in a global 3.7-Å-resolution map. This volume served as a volume template for subsequent heterogeneous refinement in a later step of EM processing.

To recover the maximum number of good particles lost during particle cleaning, each split was processed independently three times, then recombined and intersected, yielding a total of 733,085 particles. The enriched pool of 2D classes was further processed through an intensive heterogeneous refinement using three good volumes from the previous round and three low-pass filtered LF40 volumes as bait to trap the remaining bad particles. This yielded a total of 220,041 particles that provided a 3.56-Å map. An extra round of heterogeneous refinement and particle polishing with reference-based motion correction improved the resolution to 3.25 Å with 183,227 particles. At the final step, a focused three-dimensional (3D) classification with a spherical mask (15 Å, dilation radius 2 Å, soft padding width 5) centring around the binding pocket resulted in 49,481 particles (class 2). These particles were further used for non-uniform refinement (NU-refinement) with C1 symmetry imposed, yielding a global 2.88-Å-resolution map with well-defined ligand density.

We followed the same steps as described above with some modifications specific to the 2,4-D LAX3 sample. For this sample, we collected a total of 20,002 movies. The template picker yielded 11,519,397 particles, which, after a round of 2D classification, resulted in 1,470,803 particles. These particles were reextracted as pool 1 to a box size identical to the previous set-up. Following the methods described above, we managed to recover a total of 268,640 particles. Further processing of these particles led to a map resolution of 3.73 Å, utilizing 143,397 particles. During the focused 3D classification step, we centred around the binding pocket. Here, we used an iterative approach where we ran multiple 3D classifications with an increasingly tighter focus mask on the binding site, which resulted in 58,924 particles that were used for a final NU-refinement with C1 symmetry, giving a resolution of 3.43 Å.

Using a similar approach, the cryo-EM structure of IAA LAX3 was determined using two datasets, consisting of 13,772 movies (dataset 1) and 14,171 movies (dataset 2), yielding a total of 2,866,575 particles. The data underwent several processing steps, including patch motion correction, patch contrast transfer function correction and 2D classification. After class sorting and refinement, 74,645 particles were selected for the final 3D map, with a resolution of 3.21 Å. The focused classification on the binding pocket identified three particle classes, with class 0 containing the highest number of particles.

For apo LAX3 data, 10,273 movies were collected. The template picker picked 5,886,564 particles. After six rounds of 2D classification, the resulting particle stack was 2,921,542 particles. Extensive heterogeneous refinement with three volumes from the template picking and three junk volumes generated from noise resulted in a particle stack of 212,841. After 3D classification with two classes and two more heterogenous refinements, the resulting 80,204 particles were used for NU-refinement with C1 symmetry. No masks were used during final NU-refinements.

### Model building, refinement and analysis

*A. thaliana* protein sequences used in this study are available via Uniprot (https://www.uniprot.org/) with the following accession codes: AUX1 (Q96247), LAX1 (Q9LFB2), LAX2 (Q9S836) and LAX3 (Q9CA25).

The AlphaFold LAX3 structure served as the initial model for docking into the map using Chimera^39,40^. MD-based geometry fitting using molecular dynamics flexible fitting^41^ was carried out by Namdinator^42^. After this, models were manually adjusted by iterative model building in Coot^43^ combined with real-space refinement using Phenix^44^, initially with an Amber force-field MD refinement. The structure geometry was validated by monitoring MolProbity^45^, CaBLAM^46^ and Ramachandran-Z analysis^47^. Statistics of the model refinement are presented in Extended Data Table 1. Figures were prepared using PyMOL Molecular Graphics System (Schrödinger, LLC) and Chimera^40^. Conservation of residues across species was analysed using Consurf^48^. Sequence alignments were constructed with PROMALS3D^49^. Alignments were visualized using ALINE v.1.0.025^50^. Structural similarity to other protein families were identified using the DALI web server^51^. Phylogenetic analysis was performed using NGPhylogeny.fr^52^. In brief, Muscle^53^ was used for multiple sequence alignment, Noisy^54^ was used for multiple sequence alignment pruning and PhyML^55^ was used for unrooted tree generation.

### MD simulations

#### MD system preparation

The inward semi-occluded structure of LAX3 with bound IAA was prepared using Maestro 2021.4^56^. The missing loops (residues 110–114 and 342–347) were rebuilt using the Prime tool in Maestro by providing the complete sequence of the protein. The caps were added to both termini, resulting in an acetylated N terminus and an amidated C terminus. The p*K*_a_ prediction of ionizable side chains was performed by PROPKA3 (ref. ^57^). A disulfide bridge was modelled between Cys462 and Cys465 residues. As the transporter localizes on the plant plasma membrane, which exhibits a twofold difference in the solvent pH, we assigned the protonation states of the extracellular-facing residues with the reference pH 5 (thus modelling His216, His224 and His296 as positively charged), while the cytoplasmic-facing residues were modelled in a pH 7.2 environment, resulting in neutral His116, His189, His255, His319, His342 and His395 protonated on the Nε atom, and His131 protonated on the Nδ atom of the imidazole ring. Plausible protonation states of IAA and the binding site residue His247 were examined by modelling simulation systems with four different protonation patterns: IAA(0) H247(0), IAA(0) H247(+), IAA(−) H247(0) and IAA(−) H247(+). When used, the neutral form of His247 was protonated on the Nε atom of the imidazole ring.

CHARMM36 force-field parameters for the protonated and deprotonated forms of IAA were generated using CGenFF version 2021.1 (refs. ^58,59^). If modelled as neutral, the proton on the carboxyl group of IAA was assigned to the O3 atom.

LAX3 was aligned with the *z* axis using the Orientations of Proteins in Membranes (OPM) database with the Positioning of Proteins in Membranes (PPM) 3.0 web server^60^ and then inserted into the 10 × 10 nm^2^ membrane patch in an *xy* plane consisting of 1-palmitoyl-2-oleoylphosphatidylcholine lipids, containing 246 membrane lipids in total. Membrane creation and protein insertion steps were performed in CHARMM-GUI version 3.7 (refs. ^61,62^). The system was then solvated using a TIP3P water model^63^ and 0.15 M NaCl in neutralizing conditions.

#### MD simulation protocol

All simulations were run with the CHARMM36m force field (July 2022 release)^64,65^ and using the Gromacs 2024.3 simulation software^66^. All systems were created in six replicas, with each replica being individually solvated, minimized and equilibrated to ensure independent starting conditions.

The simulation systems were initially minimized using the steepest-descent algorithm until forces on each atom reached a maximum value of 1000 kJ mol^−1^ nm^−1^. The following six rounds of equilibrations were performed using the modified CHARMM-GUI protocol and with gradually decreasing position restraints on LAX3, IAA and membrane lipids.

The production runs were performed with a leapfrog integration algorithm and a 2-fs time step, producing 1 µs of simulation for each repeat. The Linear Constraint Solver (LINCS) algorithm^67^ was used to constrain the covalently bound hydrogens. The temperature of 303 K, corresponding to the plant growing conditions, was maintained using the v-rescale barostat^68^ with a temperature time constant of 1 ps and with three temperature groups (protein–ligand complex, membrane and solvent). The pressure of 1 bar was maintained with the c-rescale barostat^69^ under a semi-isotropic coupling regime and with a compressibility of 4.5 × 10^−5^ bar^−1^ and a time constant of 5 ps. The cut-off for real-space electrostatic interactions was set to 1.2 nm, while the long-range electrostatic interactions were calculated using the particle mesh Ewald algorithm^70,71^. The van der Waals interactions were considered within the 1.2-nm cut-off and with a force-switch modifier applied at 1.0 nm. Cumulatively, the production MD simulation runs generated 24 µs of sampling in total.

#### MD simulation analysis

The ligand r.m.s.d. was calculated using the Gromacs analysis suite. The r.m.s.d. of IAA was calculated using the heavy atoms of IAA in the experimentally generated structure as a reference.

The IAA poses were described in spherical coordinate system using an azimuthal (*φ*) angle (spanning the −180° to 180° range and thus describing a circle), and the polar (*θ*) angle (spanning 0° to 180°). The *φ* angle was unwrapped for the purposes of clustering analysis by taking the angle value with the lowest point density, deemed unlikely to cut through any major cluster of interest. The clustering was performed on the unwrapped *φ* and *θ* angle 2D space and using IAA poses near the binding site (that is, limited by IAA r.m.s.d. <8 Å). The resulting 54,709 points were clustered using the hierarchical-density-based spatial clustering of applications with noise (HDBSCAN) algorithm implemented in the dbscan package (version 1.2.0) in R 4.4.3. The minimum number of points per cluster was assigned to be 1,000. The representative cluster poses were extracted by identifying frames with the lowest Euclidean distance to each cluster centroid.

The number of water molecules surrounding the IAA was calculated by counting the water oxygen atoms within 6 Å distance in each frame. The ligand–protein contact analysis was performed using ProLIF software^72^, where hydrogen bonds are defined as donor–acceptor interactions bridged by a hydrogen, forming an angle ranging between 130° and 180° and with the heavy atoms within 3.5 Å of each other; the hydrophobic interactions involve two non-polar atoms that are within 4.5 Å distance. The frequencies were normalized against the highest frequency of interaction within each cluster.

### Oocyte efflux assays

For uptake assays, oocytes were injected with 50 nl containing 15 ng of cRNA and control oocytes were injected with 50 nl of water. Oocytes were incubated for 6 days in Barth’s solution (88 mM NaCl, 1 mM KCl, 2.4 mM NaHCO_3_, 10 mM HEPES, 0.33 mM Ca(NO_3_)_2_, 0.41 mM CaCl_2_ and 0.82 mM MgSO_4_) pH 7.4 at 16 °C. On the day of the experiment, oocytes were transferred to Barth’s solution pH 6, unless stated otherwise, containing indicated concentrations of ^3^H-IAA (25 Ci mmol^−1^, 1 mCi ml^−1^, Tritec) or ^3^H-2,4-D (31.2 Ci mmol^−1^,1 mCi ml^−1^, Tritec) and incubated at room temperature. The oocytes were then washed three times with Barth’s solution pH 6 and individually transferred into scintillation vials. Oocytes were lysed in 10% sodium dodecyl sulfate for 5 min. Radioactivity was determined by liquid scintillation counting in 2 ml of Rotiszint eco plus (Roth). For time-course experiments, five individual oocytes were isolated after 2, 4, 6 and 8 min. The transport rate was determined by linear regression. To correct for passive uptake, the uptake rate of water-injected oocytes was subtracted from that of LAX3-injected oocytes. For end-point measurements, substrate levels of 19–20 individual oocytes were determined after 8 min of incubation. To determine the *K*_m_, individual uptake experiments were fit to a Michaelis–Menten model, and the *V*_max_ was set to 100% for each individual replicate. All *P* values are considered significant at *P < 0.05, **P < 0.01, ***P < 0.001, ****P < 0.0001.

### Reporting summary

Further information on research design is available in the Nature Portfolio Reporting Summary linked to this article.

## Supplementary information

Reporting Summary

Supplementary Video A simulation trajectory (IAA^−^His247° repeat 1) showing the IAA unbinding event guided by Arg192 and IAA COO^−^ charge–charge interactions. The plot is simultaneously mapping the progression of the IAA heavy atom r.m.s.d. over simulation time.

## Data Availability

Atomic models have been deposited in the Protein Data Bank (PDB), and cryo-EM maps have been deposited in the Electron Microscopy Data Bank (EMDB). Apo LAX3 inward open: PDB 9QQM and EMD-53308. IAA-bound LAX3 inward semi-occluded: PDB 9H61 and EMD-51894. 2,4-D bound LAX3 inward semi-occluded: PDB 9H62 and EMD-51895. 2-NOA bound LAX3 fully occluded: PDB 9H63 and EMD-51896.

## Extended data

**Extended Data Table 1:** Statistics for cryo-EM data collection, model refinement and validation

	Inward open	Inward semi-occluded	Inward semi-occluded	Fully occluded
	Apo LAX3 pH 5.5	IAA LAX3 pH 7.5	2,4-D LAX3 pH 7.5	2-NOA LAX3 pH 7.5
	LMNG	LMNG	LMNG	LMNG
**Data Collection and processing**				
Microscope	Krios I (eBic)	Krios II (EMBION)	Krios II (EMBION)	Krios II (EMBION)
Magnification	130.000	130,000	130,000	130,000
Voltage (kV)	300	300	300	300
Electron exposure (e^−^/Å^2^)	50	59.3 and 60.0	60.0	59.70
Defocus range (μm)	0.6-2.0	0.4-2.6	0.4-2.6	0.4-2.6
Pixel size (Å)	0.645	0.647	0.647	0.647
Symmetry imposed	C1	C1	C1	C1
Collected micrographs (no.)	10.273	13,772 and 14,171	20.002	12.748
Initial particle images (no.)	5,886,564	4,432,384	1,470,803	3,465,533
Final particle images (no.)	80.204	74.645	58.924	49.481
Map resolution (Å)*	3.16	3.21	3.43	2.88
**Refinement**				
Initial model used	Alphafold	Alphafold	Alphafold	Alphafold
Map sharpening B factor (Å^2^)	96.9	111.9	105.6	90.0
Model composition				
non-hydrogen atoms	3.132	3.311	3.312	3.396
Protein residues	393	412	412	420
Ligands		IAC: 1	CFA: 1	NOA: 1
Waters	0	2	3	8
R.m.s. deviation				
Bond lenghts (Å)	0.002	0.02	0.002	0.002
Bond angles (deg)	0.419	0.506	0.412	0.564
Validation				
MolProbity score	1.25	1.28	1.24	1.34
Clashscore	3.35	4.54	4.69	4.0
Poor rotamers (%)	0.6	0.00	0.57	0.00
Rama-Z score (Whole/Helix/Loop)	2.74/2.11/1.01	0.45/0.51/0.01	2.71/2.26/0.19	1.55/1.58/-0.78
CaBLAM score (Outliers/Disfavored/Cα)	0.54/2.95/0.27	1.00/3.75/0.50	1.50/5.75/0.50	0.97/4.61/0.24
Ramachandran Plot				
Favored (%)	97.39	97.78	98.03	97.12
Allowed (%)	2.61	2.22	1.97	2.88
Disallowed (%)	0.00	0.00	0.00	0.00
Deposited model (PDB-ID)	9QQM	9H61	9H62	9H63
Deposited map (EMDB-ID)	53308	51894	51895	51896

Cryo-EM data collection, refinement and validation statistics

* Gold standard FSC with threshold of 0.143

IAC: Indole-3-acetic acid

CFA: 2,4-Dichlorophenoxyacetic acid

NOA: 2-Naphthoxyacetic acid
